# Effectiveness of mHealth on Adherence to Antiretroviral Therapy in Patients Living With HIV: Meta-analysis of Randomized Controlled Trials

**DOI:** 10.2196/42799

**Published:** 2023-01-23

**Authors:** Liang Sun, Mengbing Qu, Bing Chen, Chuancang Li, Haohao Fan, Yang Zhao

**Affiliations:** 1 College of Public Health Zhengzhou University Zhengzhou China; 2 Sanmenxia Center for Disease Control and Prevention Sanmenxia China

**Keywords:** HIV, mHealth, antiretroviral therapy, meta-analysis

## Abstract

**Background:**

The World Health Organization recommends that all adults with HIV adhere to antiretroviral therapy (ART). Good adherence to ART is beneficial to patients and the public. Furthermore, mHealth has shown promise in improving HIV medication adherence globally.

**Objective:**

The aim of this meta-analysis is to analyze the effectiveness of mHealth on adherence to antiretroviral therapy in patients living with HIV.

**Methods:**

Randomized controlled trials (RCTs) of the association between mHealth and adherence to ART published until December 2021 were searched in electronic databases. Odds ratios (ORs), weighted mean differences, and 95% CIs were calculated. This meta-analysis was performed using the Mantel-Haenszel method or the inverse variance test. We evaluated heterogeneity with the *I*^2^ statistic. If *I*^2^ was ≤50%, heterogeneity was absent, and a fixed effect model was used. If *I*^2^ was >50%, heterogeneity was present, and a random effects model was used.

**Results:**

A total of 2163 participants in 8 studies were included in this meta-analysis. All included studies were RCTs. The random effects model was used for a meta-analysis of the effects of various intervention measures compared to routine nursing; the outcome was not statistically significant (OR 1.54, 95% CI 0.99-2.38; *P*=.05). In the subgroups, only short messaging service (SMS)-based interventions significantly increased adherence to ART (OR 1.76, 95% CI 1.07-2.89; *P*=.03). Further analysis showed that only interactive or bidirectional SMS could significantly increase ART adherence (OR 1.69, 95% CI 1.22-2.34; *P*=.001). After combining the difference in CD4 cell count before and after the interventions, we concluded that there was no statistical heterogeneity among the studies (*I*^2^=0%; tau^2^=0.37; *P*=.95).

**Conclusions:**

Interactive or bidirectional SMS can enhance intervention effects. However, whether mHealth can improve adherence to ART in patients with HIV needs further study. Owing to a lack of the required significant staff time, training, and ongoing supervision, there is still much more to do to apply mHealth to the clinical use of ART for patients living with HIV.

**Trial Registration:**

PROSPERO CRD42022358774; https://www.crd.york.ac.uk/prospero/display_record.php?RecordID=358774

## Introduction

HIV is a public health issue that every country needs to address. There were an estimated 37.7 million people living with HIV at the end of 2020 [1]. The World Health Organization (WHO) recommends that all adults with HIV should adhere to antiretroviral therapy (ART) [2]. ART does not cure HIV infection but strongly suppresses viral replication within a person’s body and modifies HIV from a terminal illness to a manageable chronic disease [3]. One of the most significant factors in the effectiveness of ART is adherence [4]. Good adherence to ART is beneficial to patients and public health [5,6]. In contrast, lack of adherence increases the risk of progression to AIDS and the creation of drug-resistant strains of HIV [7]. Therefore, it is important to promote ART adherence through special techniques [8,9]. However, traditional ART adherence interventions are limited in their ability to maintain behavior modification [10]. Mobile health (mHealth) technology, which refers to the use of mobile and wireless technologies to improve health, has shown promise in improving HIV medication adherence, both globally and domestically [6,11,12]. Therefore, we performed a meta-analysis of the effectiveness of mHealth for improving adherence to ART in patients living with HIV.

## Methods

### Ethical Considerations

This paper contains no primary data obtained directly from research participants. Data obtained from previously published resources have been acknowledged with references. Ethical approval was not required.

### Protocol Registration

The review protocol was prospectively registered with PROSPERO (International Prospective Register of Systematic Reviews; CRD42022358774).

### Search Strategy

This meta-analysis was performed according to the Preferred Reporting Items for Systematic Reviews and Meta-Analyses (PRISMA) statement guidelines [13]. Searching was conducted using the electronic databases PubMed, EMbase, CINAHL, ScienceDirect, the Cochrane Library, Web of Science, and ClinicalTrials.gov to identify original articles meeting the evaluation criteria for inclusion and exclusion published until December 2021. Searching was conducted and evaluated by 2 independent reviewers. The search strategy for identifying studies included the key terms *mobile health*, *human immunodeficiency virus*, *medication adherence*, *randomized controlled trial*, and other related terms. These keywords were also combined using the OR and AND operators (Multimedia Appendix 1).

### Inclusion and Exclusion Criteria

The screening process was divided into 2 phases: a preliminary selection by title and abstract and a second phase that screened the full text of the remaining articles. Articles were included based on the following criteria: (1) they reported the results of a randomized controlled trial (RCT); (2) they included HIV-positive persons receiving antiretroviral treatment regardless of age, sex, or nationality; (3) the intervention measures included, but were not limited to, short message service (SMS) texts and voice calls; (4) an mHealth intervention was used in the experimental group with no limits on intervention frequency, time, content, or period and the control group received routine nursing at the same time to help patients improve their treatment compliance; and (5) the primary outcome was adherence to ART. This was measured directly (eg, by pill count) or indirectly (eg, by self-reporting). If the article reported the use of a variety of measurement methods, priority was given to measurement results obtained with the self-report method. The secondary outcome was CD4 cell count.

The exclusion criteria included the following: (1) the study was a duplicate, (2) the study was a systematic review or meta-analysis, (3) the study was missing outcome measures, (4) the experimental group used a variety of interventions, and (5) the control group did not receive routine nursing.

### Data Extraction

A predesigned Excel sheet was used to extract and organize the data into categories by 2 independent researchers. These data included (1) authors, (2) location, (3) publication date, (4) intervention details (ie, intervention mode and duration), (5) outcome measures, including ART adherence and CD4 cell counts, and (6) risk of bias.

### Risk of Bias and Quality Assessment

**Figure 1:**
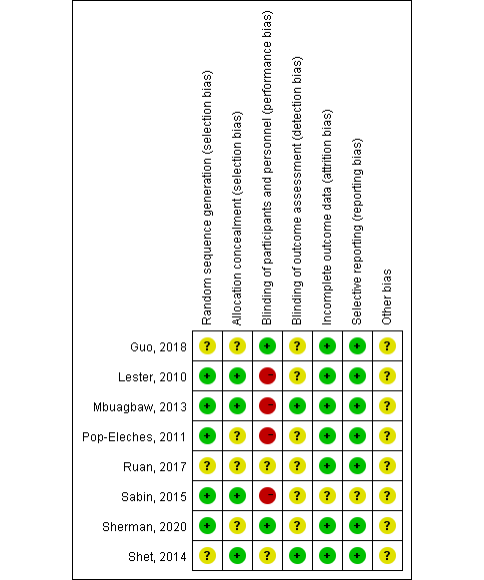
Risk of bias of the studies included in the meta-analysis. Green indicates a low risk of bias, yellow indicates an unclear risk of bias, and red indicates a high risk of bias.

Two of the authors assessed the risk of bias using RevMan (version 5.4; Cochrane Collaboration); the results are summarized in Figure 1. The Cochrane Collaboration’s Risk of Bias tool was also used to assess quality and risk of bias. This tool assesses bias in 7 domains: random sequence generation (for selection bias), concealment of the allocation sequence (for selection bias), blinding of participants and personnel (for performance bias), blinding of outcome assessment (for detection bias), incomplete outcome data (for attrition bias), selective outcome reporting (for reporting bias), and other biases. Studies are assigned a low risk of bias, an unclear risk of bias, or a high risk of bias.

### Data Analysis

The meta-analysis was conducted using RevMan. Measures of effect are presented as odds ratios (ORs) with the 95% CI. For continuous data, we calculated the sample-size weighted mean difference (WMD). This meta-analysis was performed using the Mantel-Haenszel method or the inverse variance test. We evaluated heterogeneity with the *I*^2^ statistic. If *I*^2^ was ≤50%, heterogeneity was absent, and a fixed effect model was used. If *I*^2^ was >50%, heterogeneity was present, and a random effects model was used.

## Results

### Study Selection Process

**Figure 2:**
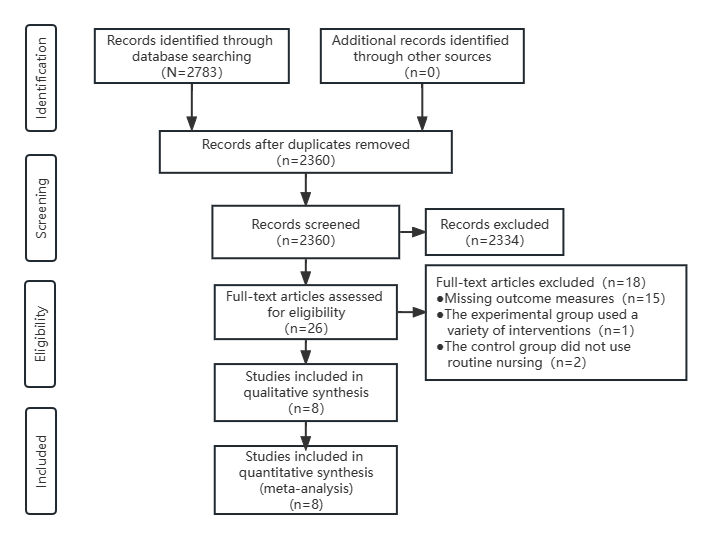
Flow chart of study selection for the meta-analysis.

The search strategy identified 2783 articles from the electronic databases. In total, 423 articles were excluded because of duplication. We screened the titles and abstracts of the remaining articles and included 26 for full-text review based on the inclusion and exclusion criteria. Of these 26 studies, 8 met the inclusion criteria, and 18 studies were excluded: 15 because they were missing outcome measures, 1 because a variety of interventions were used in the experimental group, and 2 because they did not use routine nursing in the control group. Therefore, 8 studies were selected for the current meta-analysis [4,9,14-19] (Figure 2).

### Study Characteristics

The characteristics of the studies are summarized in Table 1. All included studies were RCTs. A total of 2163 participants in 8 studies were included in this meta-analysis. Except for the study by Mbuagbaw et al [19], all participants in the studies were aged 18 years or older. Study duration ranged between 1 and 12 months. SMS was used as the basis for the intervention in 6 studies. One of these studies used an mHealth intervention program that included text messages and WeChat. The remaining studies used voice calls.

**Table 1:** Characteristics of the included studies. All studies used routine nursing in the control group.

First author, year	Location	Participants, n (age, years)	Recruitment period (duration)	Intervention	Outcome measures
Ruan, 2017 [4]	Hengyang, China	100 (≥18)	Mar 2013-Mar 2014 (6 months)	SMS^a^	ART^b^ adherence measured by VAS^c^, Community Programs for Clinical Research on AIDS Antiretroviral Medication Self-Report, and CD4 cell count
Guo, 2018 [14]	South China	53 (≥18)	Oct 2016-Mar 2017 (3 months)	SMS and WeChat	CD4 cell count
Shet, 2014 [15]	South India	631 (18-60)	Jul 2010-Aug 2011 (6 months)	Customized motivational voice call	ART adherence measured by pill count
Sherman, 2020 [16]	South Florida, US	94 (>18)	Sept 2011-Apr 2014 (3-6 months)	Automated 1-way medication reminders delivered via SMS	ART adherence measured by VAS and CD4 cell count
Lester, 2010 [17]	Kenya	538 (>18)	May 2007-Oct 2008 (12 months)	SMS	ART adherence measured by self-report
Sabin, 2015 [9]	Guangxi, China	119 (≥18)	Dec 2012-Apr 2013 (6 months)	SMS	ART adherence measured by a medication device
Pop-Eleches, 2011 [18]	Kenya	428 (>18)	Jun 2007-Aug 2008 (3-12 months)	SMS	ART adherence measured by a medication event monitoring system and CD4 cell count
Mbuagbaw, 2013 [19]	Cameroon	200 (≥21)	Nov 2010-Dec 2010 (3-6 months)	Weekly standardized motivational text message	VAS and self-reported adherence

^a^SMS: short message service.

^b^ART: antiretroviral therapy.

^c^VAS: visual analog scale.

### Meta-analysis

#### Medication Adherence

**Figure 3:**
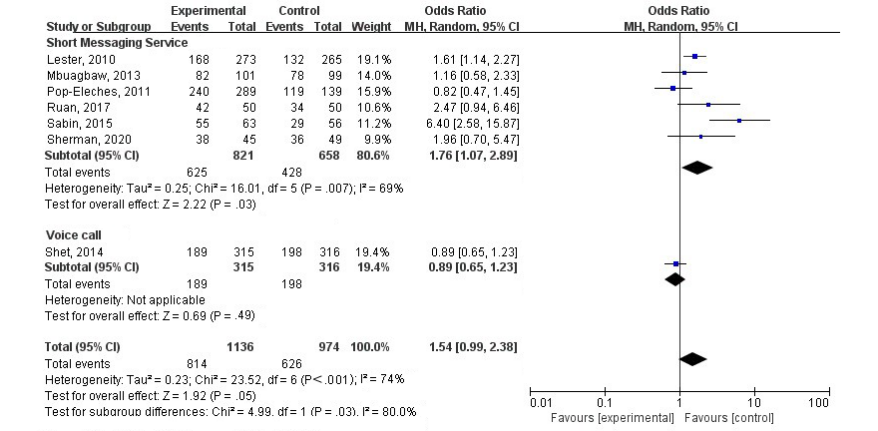
Forest plot of odds ratios with 95% CIs for the effect of various intervention measures and routine nursing on adherence to antiretroviral therapy. MH: Mantel-Haenszel method.

Adherence to ART was measured as a primary outcome in 7 studies. The method and frequency of measuring adherence varied across the studies. The details are listed in Table 1. A random effects model was used for a meta-analysis of the effects of various intervention measures and routine nursing; the outcome was not statistically significant (OR 1.54, 95% CI 0.99-2.38; *P*=.05). There was also evidence of heterogeneity among the studies (*I*^2^=74%; tau^2^=0.23; *P*<.001; Figure 3). In the subgroups, only SMS interventions significantly increased adherence to ART (OR 1.76, 95% CI 1.07-2.89; *P*=.03).

#### Medication Adherence With SMS Intervention

**Figure 4:**
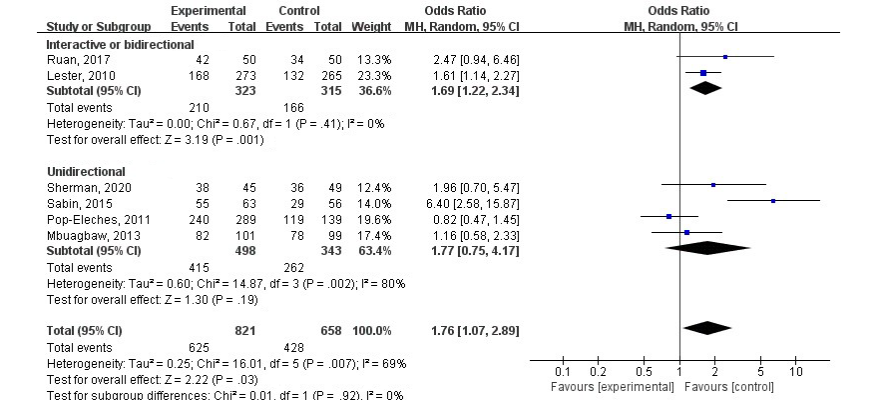
Forest plot of odds ratios with 95% CIs for the effect of short message service interventions on adherence to antiretroviral therapy. MH: Mantel-Haenszel.

Six studies were included in a meta-analysis of the effect of SMS on adherence to ART; this showed that SMS could improve adherence (OR 1.76, 95% CI 1.07-2.89; *P*=.03) but also revealed considerable heterogeneity among the included studies (*I*^2^=69%; tau^2^=0.25; *P*=.007; Figure 4). In the subgroups, only interactive or bidirectional SMS interventions could significantly increase ART adherence (OR 1.69, 95% CI 1.22-2.34; *P*=.001). However, these studies did not show statistical heterogeneity (*I*^2^=0%; tau^2^=0.00; *P*=.41). A subgroup analysis of studies with unidirectional SMS interventions showed no statistical heterogeneity, but the analysis also showed a lack of effect in improving adherence (Figure 4).

#### CD4 Cell Count

**Figure 5:**
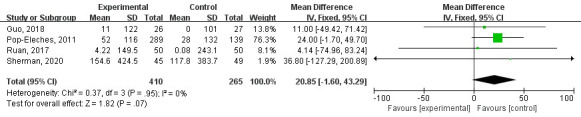
Forest plot of pooled odds ratios with 95% CIs for the effect of mHealth interventions on CD4 cell count. IV: inverse variance.

Four studies reported CD4 cell count as a secondary outcome of medication adherence. Combining the differences in CD4 cell count before and after the interventions revealed no statistical heterogeneity among the studies (*I*^2^=0%; tau^2^=0.37; *P*=.95; Figure 5). Meta-analysis showed that CD4 cell count measures after mHealth interventions revealed no significant difference in medication adherence among HIV patients compared with routine nursing (WMD=20.85, 95% CI 1.60-43.29; *P*=.07; Figure 5). Only 2 studies reported viral load as an outcome to evaluate the intervention effect, so we did not perform a meta-analysis.

## Discussion

### Principal Findings

A total of 2163 participants in 8 studies were included in this meta-analysis. The main result of the meta-analysis was that the pooled OR was 1.54. However, the outcome was not statistically significant, and there was considerable heterogeneity among the studies (*I*^2^=74%). Within-study heterogeneity reduces study robustness and relevance [20]. Our current results are different from those of a 2015 study [21]. That study found that mHealth interventions did seem to have been beneficial. We speculate that one of the possible reasons for this difference is that the number of articles included in our study was limited. Also, the different studies used a variety of methods to measure results. However, compared with the 2015 study, this meta-analysis combined the outcomes (separately for primary and secondary outcomes) to increase the reliability of the results.

A subgroup analysis showed that SMS interventions improved adherence to ART. Further analysis suggested that interactive or bidirectional SMS interventions could enhance intervention effects. This result matches that of a 2014 study [22]. Interactive or bidirectional SMS could increase medication adherence by enhancing engagement and the patient-provider relationship. Follow-up research could further study the content, time, and frequency of text messages. In this study, due to limitations arising from incomplete reporting of the relevant content, these aspects of the intervention were not analyzed.

CD4 cell count and viral load are good indicators of treatment success [23]. Several previous reviews did not obtain the same results as this study for CD4 cell count. One study [22] showed that compared to a control group, a group receiving text message–based support was more likely to maintain an adherence threshold at follow-up and meet the clinical goal of a higher CD4 cell count. However, another study [24], like ours, did not obtain this result. Our meta-analysis of the 4 studies that reported CD4 cell count showed considerable heterogeneity and no significant pooled mean difference. This may be attributable to the heterogeneity of the studies. We did not perform a meta-analysis of studies that measured viral load, as there was an insufficient number of these studies.

The WHO recommends mHealth strategies for improving ART adherence [25]. At the same time, against the background of the coronavirus epidemic, telemedicine has gradually continued to develop [26]. Currently, 95% of people use mobile phones and 77% of people use smartphones in different parts of the world [27]; mHealth has shown promise in increasing the accessibility of self-management interventions [28] and improving HIV health outcomes [6]. At the same time, research has indicated that patients living with HIV are interested in mobile apps to support HIV self-management [29]. However, most current mHealth interventions lack functionality, offering only medication reminders [28] or voice calls. Therefore, more comprehensive mHealth interventions that address multiple self-management needs of patients living with HIV are needed [30]. At the same time, the implementation of ART interventions in real-world clinical settings has been severely limited by a lack of the resources required to initiate and maintain the interventions [31], such as staff time, training, and ongoing supervision. Future research should focus on how to apply personalized mHealth interventions to the management of patients living with HIV.

### Limitations

There are several limitations of the current review. The interventions used in the included studies differed in form and frequency. At the same time, these studies used diverse methods for measuring their primary outcomes. This may have produced bias. The robustness and relevance of results increase with the number of distinct outcome measures that show the same result [20]. However, few of the included studies reported CD4 cell count or viral load, and our analysis of these outcomes is thus insufficient.

### Conclusion

Interactive or bidirectional SMS interventions can enhance intervention effects. However, whether mHealth can improve adherence to ART in patients with HIV is a question that needs further study. Owing to a lack of staff time, training, and ongoing supervision, there is still much work to be done to use mHealth in the clinic for ART adherence among patients living with HIV.

## Appendix

Multimedia Appendix 1 Keywords.

## Abbreviations

ART: antiretroviral therapy
mHealth: mobile health
OR: odds ratio
PRISMA: Preferred Reporting Items for Systematic Reviews and Meta-Analyses
RCT: randomized controlled trial
SMS: short message service
VAS: visual analog scale
WMD: weighted mean difference
