# Supplementary material for: Effectiveness of mHealth on Adherence to Antiretroviral Therapy in Patients Living With HIV: Meta-analysis of Randomized Controlled Trials
Source: JMIR Mhealth Uhealth. 2023 Jan 23;11:e42799. doi: 10.2196/42799 (PMC9903184; doi:10.2196/42799)
Supplement: Multimedia Appendix 1 [file mhealth_v11i1e42799_app1.docx]

Supplementary File 1. Search strategy

#1 (Mobile Health) OR (Health, Mobile) OR (mHealth) OR (mHealth) OR (Telehealth) OR (eHealth)

#2 (Human Immunodeficiency Virus) OR (Immunodeficiency Virus, Human) OR (Immunodeficiency Viruses, Human) OR (Virus, Human Immunodeficiency) OR (Viruses, Human Immunodeficiency) OR (Human Immunodeficiency Viruses) OR (Human T Cell Lymphotropic Virus Type III) OR (Human T-Cell Lymphotropic Virus Type III) OR (Human T-Cell Leukemia Virus Type III) OR (Human T Cell Leukemia Virus Type III) OR (LAV-HTLV-III) OR (Lymphadenopathy-Associated Virus) OR (Lymphadenopathy Associated Virus) OR (Lymphadenopathy-Associated Viruses) OR (Virus, Lymphadenopathy-Associated) OR (Viruses, Lymphadenopathy-Associated) OR (Human T Lymphotropic Virus Type III) OR (Human T-Lymphotropic Virus Type III) OR (AIDS Virus) OR (AIDS Viruses) OR (Virus, AIDS) OR (Viruses, AIDS) OR (Acquired Immune Deficiency Syndrome Virus) OR (Acquired Immunodeficiency Syndrome Virus) OR (HTLV-III))

#3 (medication) OR (treatment))

#4 (adherence) OR (compliance)

#5 (randomized controlled trial) OR (RCT)

#6 #1 and #2 and #3 and #4 and #5
